# Exploring the Differential Expression of a Set of Key Genes Involved in the Regulation and Functioning of the Stomach in the Post-Weaned Pig

**DOI:** 10.3390/vetsci10070473

**Published:** 2023-07-20

**Authors:** Dillon P. Kiernan, John V. O’Doherty, Kathryn Ruth Connolly, Marion Ryan, Torres Sweeney

**Affiliations:** 1 School of Veterinary Medicine, University College Dublin, Belfield, D04 W6F6 Dublin, Ireland; dillon.kiernan@ucdconnect.ie; 2 School of Agriculture and Food Science, University College Dublin, Belfield, D04 W6F6 Dublin, Ireland; john.vodoherty@ucd.ie (J.V.O.); ruth.connolly@ucdconnect.ie (K.R.C.); marion.ryan@ucd.ie (M.R.)

**Keywords:** monogastric, acid secretion, fundic gland region, pyloric gland region, cardiac gland region, cardiac-to-oxyntic transition, glandular regions

## Abstract

**Simple Summary:**

On commercial pig farms, weaning occurs prematurely at approximately four weeks of age, when the pig’s digestive and immune systems have not yet matured. The post-weaning period is often associated with poor performance, diarrhoea and even mortality. One area which has been somewhat overlooked, in terms of improving the health and performance of the post-weaned pig, is the stomach. The stomach’s acidic environment acts as one of the first lines of defence against ingested pathogens and plays a key role in ensuring optimal activity of certain digestive enzymes. The post-weaned pig’s poorly developed enzyme and acid secretory capacity leaves the pig more susceptible to pathogens and reduces its protein digestion capabilities. Improving the stomach’s functioning has the potential to enhance the health and performance of the post-weaned pig. However, to date, there have been minimal studies characterizing the different regions of the pig’s stomach, and more precisely the gene expression patterns in these regions. An advancement in the knowledge and understanding of the regions of the pig’s stomach would allow future researchers to more effectively target improved stomach functioning. The present study characterizes the gene expression patterns in the glandular regions (cardiac, fundic and pyloric) of the stomach, enhancing the understanding of the functions of each of these regions.

**Abstract:**

Despite playing a key role in digestion, there is only a broad characterization of the spatiotemporal development of the three glandular regions of the stomach (cardiac, fundic and pyloric) in the weaned pig. Hence, the objective of this experiment was to explore the differential expression (DE) of a panel of key genes within the three glandular regions of the stomach. Eight pigs were sacrificed at d 8 post-weaning, and three mucosal samples were collected from each stomach’s glandular regions. The expression of a panel of genes were measured using QPCR. The true cardiac gland region was characterized by increased expression of *PIGR*, *OLFM4*, *CXCL8* and *MUC2* relative to the two other regions (*p* < 0.05). The fundic gland region was characterized by increased expression of *ATP4A*, C*LIC6*, *KCNQ1*, *HRH2*, *AQP4*, *HDC*, *CCKBR*, *CHIA*, *PGA5*, *GHRL* and *MBOAT4* compared to the two other regions (*p* < 0.05). The pyloric gland region was characterized by exclusive expression of *GAST* (*p* < 0.05). A transition region between the cardiac and fundic region (cardiac-to-oxyntic transition) was observed with a gene expression signature that resembles a cross of the signatures found in the two regions. In conclusion, unique gene expression signatures were identifiable in each of the glandular regions, with a cardiac-to-oxyntic transition region clearly identifiable in the post-weaned pigs’ stomachs.

## 1. Introduction

To meet economic demand on commercial pig farms, weaning occurs prematurely at approximately four weeks of age, when the pig has an immature gastric acid and enzyme secretory capacity [1]. The number of parietal cells, i.e., H^+^/K^+^-ATPase positive cells which are crucial for H^+^ secretion, increases steadily with age from birth to weaning, with a significant increase observed from 7 to 14 days post-weaning [2,3]. The low abundance and immaturity of parietal cells in the pig’s stomach at this time means the pig has a limited capacity to maintain low gastric pH. Hence, the suckling pig is reliant on lactic acid and acetate, produced from the breakdown of lactose by bacteria in the stomach, to maintain gastric acidity [4,5]. In commercial weaning, the pigs diet is prematurely switched from sow’s milk to solid feed, resulting in an abrupt reduction in dietary lactose [6], with immediate implications for stomach pH. The acid-binding capacity of the mineral content of the solid feed is high, leading to further increases in the stomach pH [7,8]. This lack of lactose substrate combined with an increase in buffering from the feed exacerbates the limited capacity of the weaned pig to secrete HCl, resulting in an increase in gastric pH during the post-weaning period. This leaves the pig more susceptible to pathogens and reduces the activity of proteolytic enzymes, such as pepsin, crucial for protein digestion [9].

Strategies that support the development of the pre-weaned pigs’ stomachs with regard to increasing acid secretion and enzyme secretory capacity have been somewhat overlooked, and there are only a limited number of studies that analyse the effects of dietary modulation on the development or functioning of the stomach in pigs [10,11,12,13,14]. In fact, on a broader level, there are few studies exploring the spatiotemporal distribution of specialized cells and associated marker gene expression during the maturation of the gut in the weaned pig. The fully differentiated stomach consists of functionally different regions, which can be divided into nonglandular (oesophageal region) and glandular (cardiac, fundic and pyloric gland regions). There is a lack of concordance in the spatiotemporal mapping of these regions in the literature, and even less information on the development of these specialized regions as they pertain to their anatomical locations as the pig matures [15,16,17]. During embryonic development, the stomach obtains its distinct shape and expected gross appearance between 20 and 35 days post-fertilization, with several signalling pathways [18] and transcription factors regulating foregut patterning, stomach specification and gastric regionalization directing this process [19]. The complexity of events that contribute to both the migration and differentiation of specialized cells may mean that there is inherent variation in the regional specialization of the neonate stomach from birth [18]. The lack of descriptive studies in the literature and the variation in labelling from one study to another makes the stomach region difficult to sample accurately for further downstream analysis. Molecular and histological characterization are promising with regard to mapping subtle variations in specialized regions of the stomach, as known marker genes (associated with key cell types) can be pinpointed irrespective of the exact anatomical location. 

In pigs, maturation of the neonate stomach could be enhanced through the inclusion of novel feed ingredients and/or feeding techniques [1,11,20]. However, to target the stomach there must first be an increase in the level of knowledge surrounding the distinct regions of the stomach and the role they play in its functioning and development. Hence, the objectives of this study were firstly to explore the differential expression of a panel of key genes involved in the regulation and functioning of the cardiac, fundic and pyloric gland regions; and secondly to assess the differential gene expression within each of the regions to identify the significance of precise sampling location in the post-weaned pig’s stomach.

## 2. Materials and Methods

All experimental procedures described in the present study were approved under University College Dublin Animal Research Ethics Committee (AREC-20-22-ODoherty) and were conducted in accordance with Irish legislation (SI no. 543/2012) and the EU directive 2010/63/EU for animal experimentation.

### 2.1. Experimental Design and Diets

Twenty-four pigs (progeny of meatline Hermitage boar (Sion Road, Kilkenny, Ireland) × (Large White × Landrace sow)) were sourced from a commercial farm at weaning with an average body weight of 7.5 ± 0.65 kg (30 days of age). The pigs were housed in groups of three (1.68 × 1.22 m). The pigs were fed a standard starter diet for the duration of the experiment (Table 1). The ambient environmental temperature within the house was thermostatically controlled at 30℃ for the first 7 days and then reduced by 2 °C after week 1. Humidity was controlled at 65%. Feed was provided in meal form in two-space feeders equipped with nipple drinkers for water. 

### 2.2. Sample Collection

**Figure 1:**
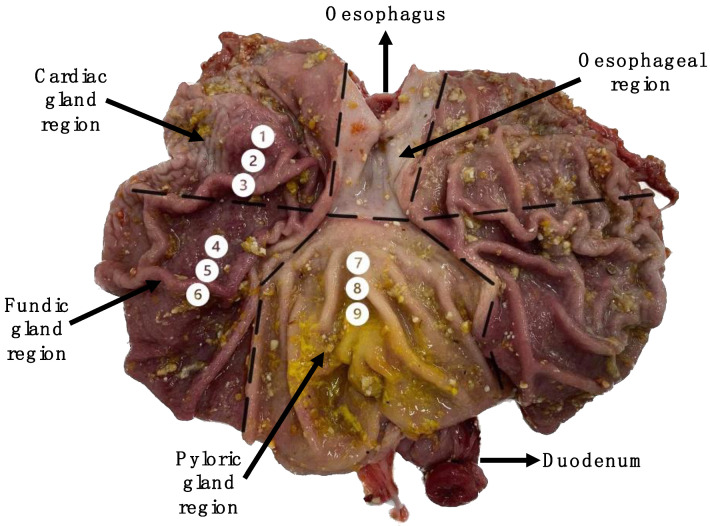
Labelled image of the pig’s stomach exposed along the greater curvature of the stomach. Mucosal sampling sites (1–9) are highlighted.

At eight days post-weaning, one pig/pen (*n* = 8) (38 days of age) received a lethal injection with pentobarbitone sodium (Euthatal Solution, 200 mg/mL; Merial Animal Health) at a rate of 0.71 mL/kg BW to the cranial vena cava to humanely sacrifice the animals. Euthanasia was completed by a competent person in a room separate from other pigs. The pigs were not fasted prior to sacrifice. The stomach was dissected from the gastrointestinal tract at the oesophagus and the duodenum. The stomach was weighed, and the pH of the stomach contents was measured by inserting a pH probe meter (MettlerToldedo, FiveEasy Plus) into the centre of the stomach lumen. The stomach was dissected along the greater curvature of the stomach, the contents were removed and the empty stomach was weighed. The pH of the mucosa was measured using litmus paper at locations 2, 5 and 8 in Figure 1. The stomach lining was gently rinsed with sterile phosphate-buffered saline (PBS). Three mucosal samples (1 cm) were collected from each of the glandular regions: the cardiac gland region (1–3); the fundic gland region (4–6); and the pyloric gland region (7–9) (See Figure 1 for sampling locations). The tissues were rinsed in PBS, stripped of the overlying smooth muscle and stored in RNAlater^®^ solution (5 mL) overnight at 4 °C. The RNAlater^®^ was removed twenty-four hours later and the samples were stored at −80 °C. 

### 2.3. Gene Expression

#### 2.3.1. RNA Extraction and cDNA Synthesis 

Total RNA was extracted from 100 mg tissue using TriReagent (Sigma-Aldrich, St. Louis, MO, USA) following the manufacturer’s instructions and then further purified using the GenElute™ Mammalian Total RNA Miniprep kit (Sigma-Aldrich, St. Louis, MO, USA). This protocol incorporated a DNase step using an on-column DNase 1 Digestion set (Thermo Scientific, Waltham, MA, USA) and the purity and quantity was assessed by determining the ratio of the absorbance at 260 nm and 280 nm on a Nanodrop-ND1000 spectrophotometer (Thermo Scientific). Total RNA (2 µg) was reverse-transcribed using a High-Capacity cDNA Reverse Transcription Kit (Applied Biosystems) and oligo (dT) primers in a final reaction volume of 40 μL. The synthesized cDNA was then diluted to a final volume of 400 μL, using nuclease-free water. 

#### 2.3.2. Target Selection

A review of the literature on the stomach functioning across several different species led to the identification of targets, capturing a range of stomach functions, as detailed in Table 2. These included genes involved in the functioning of cell types within each region of the stomach of the pig [11,16,21]. Optimal reference genes were selected using the GeNorm algorithm in qBase PLUS software (BioGazzelle, Ghent, Belgium). 

#### 2.3.3. Quantitative PCR

The quantitative PCR (qPCR) reaction mix (20 μL) contained GoTaq qPCR Syber Green Master Mix (10 μL) (Promega, Madison, WI, USA), forward and reverse primers (1.2 μL of a 5 μM mix) giving a final concentration of 300 nM/RXN, nuclease-free water (3.8 μL) and cDNA (5 μL) equivalent to 25 ng total RNA. All qPCR reactions were performed in duplicate on the 7500 ABI Prism Sequence Detection System (Applied Biosystems, Foster City, CA, USA). The cycling conditions included a denaturation step of 95 °C for 10 min followed by 40 cycles of 95 °C for 15 s and 60 °C for 1 min. Primers were designed using Primer Express™ software (Applied Biosystems, Foster City, CA, USA) and synthesized by Eurofins (Milton Keynes, UK). The geometric mean of the optimal reference genes ACTB and RPL27 was used to normalise target expression when analysing the DE between the regions. Normalised relative quantities were obtained using the qbase PLUS software (BioGazelle, Ghent, Belgium). The accession numbers, primers sequences and amplicon lengths are given in Table 2. 

#### 2.3.4. Statistical Analysis

The gene expression data were initially checked for normality using the univariate procedure (PROC UNIVARIATE) on Statistical Analysis Software (SAS) (SAS Institute, Cary, NC, USA) and they were transformed if necessary. The data were then analysed using the general linear model procedure (PROC GLM) on SAS (version 9.4, SAS Institute). 

The DE of individual genes between the cardiac, fundic and pyloric gland regions and the DE between location points in each region was assessed. For the DE of individual genes between the cardiac, fundic and pyloric gland regions, the model included all three mucosal sample locations from each pig as individual entries for that region. Contrast statements were used to separate means. For the analysis of the DE between location points within the tissues, each tissue was analysed separately. For the analysis of function, the average expression of the selected functionally similar genes was calculated per location for each pig, and this average value was treated as a single entry. 

The probability level that denoted significance was *p* < 0.05. The probability level that denoted a tendency was *p* < 0.1. Correlograms representing Pearson correlation mapped to the first principal component were generated for each of the three regions using the package ‘Corrplot’ [22] within R [23].

## 3. Results

### 3.1. Stomach Weights 

The average full stomach weight was 452 g (SD 81 g), while the average empty stomach weight was 108 g (SD 9 g). The average stomach content weight was calculated as 344 g (SD 73 g) (sum of (individual full stomach weight—individual empty stomach weight)/number of pigs).

### 3.2. Stomach pH

The average pH of stomach content was 2.52 (SD 0.6). The average mucosal pH in location 2 (the cardiac gland region) was 4 (SD 0.5), location 5 (the fundic gland region) was 3 (SD 1.3) and location 7 (the pyloric gland region) was 3.5 (SD 1.2).

### 3.3. Comparing Gene Expression between the Cardiac, Fundic and Pyloric Regions 

#### 3.3.1. The Cardiac vs. Fundic vs. Pyloric Gland Regions 

Genes that are DE between the cardiac, fundic and pyloric gland regions are presented in Table 3, while differentially enriched functions are presented in Table 4. The entire table of average gene expression values per location in the stomach can be seen in Appendix A Table A1.

##### The Cardiac Gland Region

Five genes had greater expression in the cardiac gland region compared to the fundic gland region: *CXCL8*, *PIGR*, *OLFM4*, *MUC2* and *TLR2.* Five genes had greater expression in the cardiac gland region compared to the pyloric gland region: *GHRL*, *MBOAT4*, *CXCL8*, *PIGR* and *MUC2*. *KCNQ1*, *HDC* and *TLR4* tended to have a greater expression in the cardiac gland region compared to the pyloric gland region. When genes were grouped and analysed by function, acid secretion and ghrelin production were enriched in the cardiac gland region compared to the pyloric gland region.

##### The Fundic Gland Region

Seventeen genes had greater expression values in the fundic gland region compared to the cardiac gland region: *AQP4*, *ATP4A*, *CLIC6*, *HRH2*, *KCNQ1*, *CHIA*, *PGA5*, *CCKBR*, *GHRL*, *PCSK1*, *CBLIF*, *HDC*, *MUC1*, *MUC5AC*, *MUC6*, *TLR3* and *TLR5. MBOAT4* tended to have greater expression in the fundic gland region compared to the cardiac gland region. Eleven genes had greater expression in the fundic gland region compared to the pyloric gland region: *AQP4*, *ATP4A*, *CLIC6*, *HRH2*, *KCNQ1*, *CHIA*, *PGA5*, *CCKBR*, *GHRL*, *MBOAT4* and *HDC. PCSK1* tended to have a greater expression in the fundic compared to the pyloric gland region. When genes were grouped and analysed by function, acid secretion, ghrelin production and digestive enzymes genes were enriched in the fundic gland region compared to the cardiac and pyloric gland regions. Toll-like receptors were enriched in the fundic gland region compared to the cardiac gland region. Mucus production tended to be enriched in the fundic gland region compared to the cardiac gland region.

##### The Pyloric Gland Region

Eight genes had greater expression in the pyloric gland region compared to the cardiac gland region: *GAST*, *PCSK1*, *CBLIF*, *MUC5AC*, MUC*6*, *SST*, *TLR3* and *TLR5.* Five genes had greater expression in the pyloric gland region compared to the fundic gland region: *GAST*, *CBLIF*, *OLFM4*, *SST* and *TLR2. PIGR* and *TLR3* tended to have greater expression in the pyloric gland region compared to the fundic gland region. When genes were grouped and analysed by function, mucus production and toll-like receptor genes were enriched in the pyloric gland region compared to the cardiac gland region.

### 3.4. Comparing Gene Expression within the Cardiac, Fundic and Pyloric Regions 

#### 3.4.1. The Cardiac Gland Region (Location 1 vs. Location 2 vs. Location 3)

The data on differential expression (DE) and enriched functions between the three sampling locations within the cardiac region are presented in Table 5 and Table 6, respectively. *ATP4A*, *CLIC6*, *HRH2*, *CHIA*, *CCKBR*, *CBLIF* and *AQP4* expression was greater in location 3 compared to location 1. *PGA5*, *HDC* and *PCSK1* tended to be greater in location 3 compared to location 1. *OLFM4* and *PIGR* expression was greater in location 1 compared to location 3. *MUC2* tended to be greater in location 1 compared to location 3. *CBLIF* tended to be greater in location 3 compared to location 2. When genes were grouped and analysed by function, acid secretion and digestive enzyme production were enriched in location 3 compared to location 1.

#### 3.4.2. The Fundic Gland Region (Location 4 vs. Location 5 vs. Location 6)

There was no differential expression between locations in the fundic gland region for any individual genes or differentially enriched functions (*p* > 0.05). 

#### 3.4.3. The Pyloric Gland Region (Location 7 vs. Location 8 vs. Location 9)

There was no differential expression between locations within the pyloric gland region for any individual genes or differentially enriched functions (*p* > 0.05).

### 3.5. Gene Expression Dynamics per Tissue 

**Figure 2:**
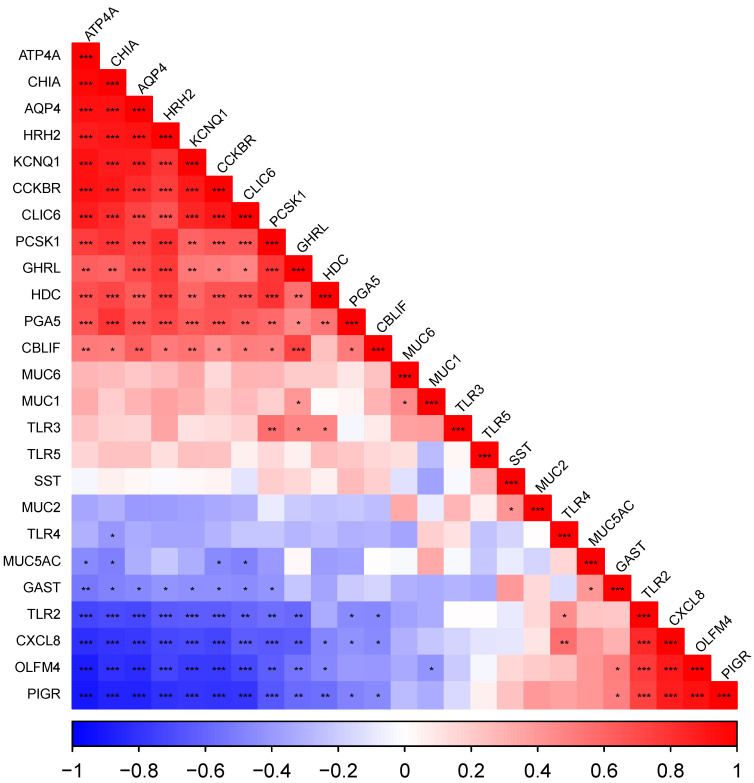
Correlogram representing Pearson correlations ordered by loadings to the first principal component in the cardiac gland region (sample locations 1, 2 and 3). Level of significance indicated by asterisks (* *p* = 0.05, ** *p* = 0.01, *** *p* = 0.001).

**Figure 3:**
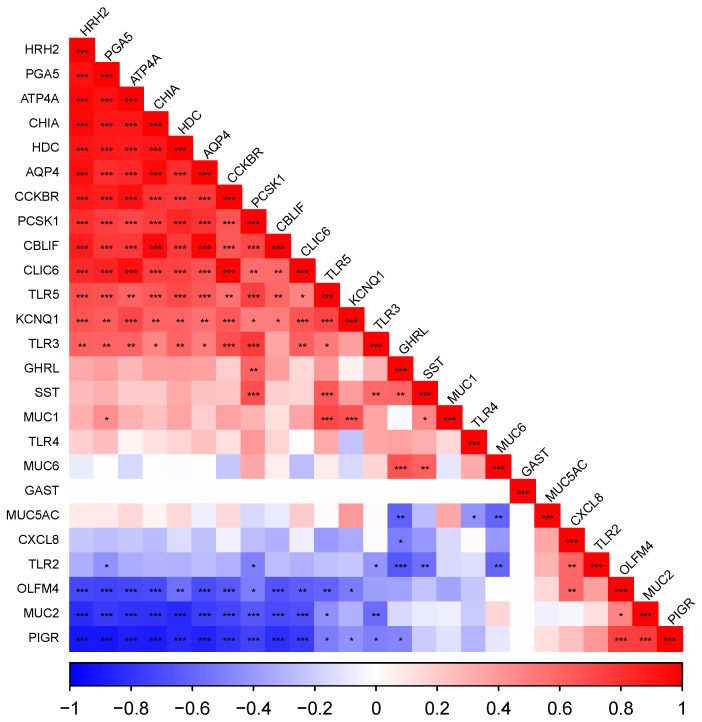
Correlogram representing Pearson correlations ordered by loadings to the first principal component in the fundic gland region (sample locations 4, 5 and 6). Level of significance indicated by asterisks (* *p* = 0.05, ** *p* = 0.01, *** *p* = 0.001).

**Figure 4:**
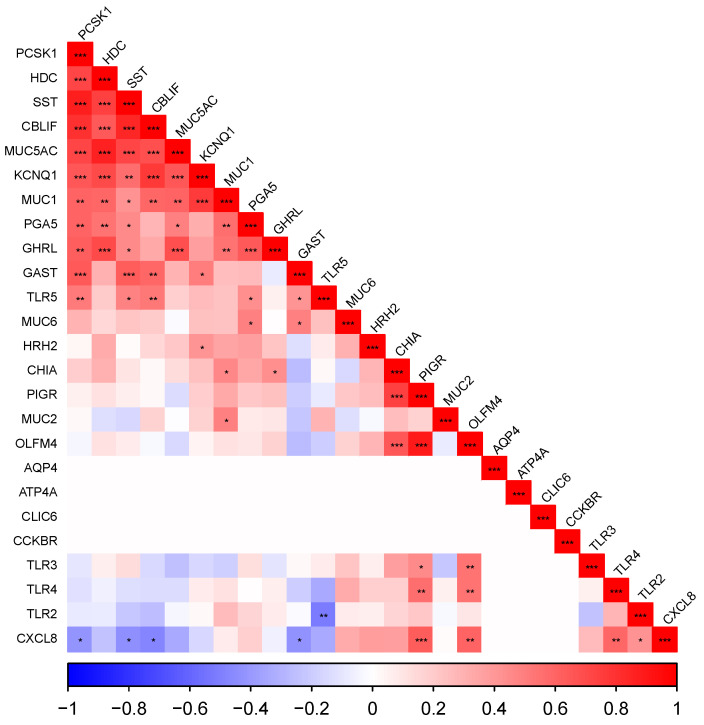
Correlogram representing Pearson correlations ordered by loadings to the first principal component matrix for gene expression in the pyloric gland region (sample locations 7, 8 and 9). Level of significance indicated by asterisks (* *p* = 0.05, ** *p* = 0.01, *** *p* = 0.001).

Correlograms representing Pearson correlations between all genes measured, ordered by their loadings to the first principal component (FPC), are presented for cardiac (Figure 2), fundic (Figure 3) and pyloric (Figure 4) gland regions. Level of significance is indicated by asterisks (* = 0.05, ** = 0.01, *** = 0.001).

#### 3.5.1. The Cardiac Gland Region

Most notably, there was a strong positive correlation between the acid secretion genes (*ATP4A*, *CLIC6*, *HRH2*, *KCNQ1*), digestive enzyme genes (*CHIA*, *PGA5*), *HDC*, *PCSK1*, *CCKBR*, *TLR3* and *CBLIF* expression, all of which were negatively correlated with the mucosal defence genes (*PIGR*, *OLFM4*, *MUC2*) which were also strongly correlated with each other.

#### 3.5.2. The Fundic Gland Region

There was a strongly positive correlation between the acid secretion genes (*ATP4A*, *CLIC6*, *HRH2*, *KCNQ1*), digestive enzyme genes (*CHIA*, *PGA5*), *HDC*, *PCSK1*, *CCKBR*, *GHRL* and *AQP4* (*p* < 0.05), all of which were negatively correlated with mucosal defence genes (*PIGR*, *OLFM4*) and immune genes (*CXCL8* and *TLR2*) (which were positively correlated with each other (*p* < 0.05)). 

#### 3.5.3. The Pyloric Gland Region

There was positive correlation between *PCSK1*, *HDC*, *SST*, *CBLIF*, *MUC5AC*, *KCNQ1* and *MUC1* (*p* < 0.05). The inflammatory cytokine *CXCL8* was positively correlated with *TLR2* and *TLR4* (*p* < 0.05) and negatively with *CBLIF*, *PCSK1*, *SST* and *GAST* (*p* < 0.05). 

## 4. Discussion

The expression patterns of key genes involved in the regulation and functioning of the stomach of the post-weaned pig were compared across the cardiac, fundic and pyloric regions, with substantial variation in most genes observed across the tissues. To the best of our knowledge, this is the first study to compare the expression patterns of key genes involved in the functioning of the stomach in the cardiac gland region in the pig. This analysis revealed the extent of the size of the cardiac-to-oxyntic transition zone, which is located between the cardiac and fundic gland regions. It also suggests that tissue sampling from the cardiac gland region should be subdivided into the “true cardiac gland region” and the “cardiac-to-oxyntic transition region”. 

In the current study, the expression of the acid-secreting genes *ATP4A*, *CLIC6* and *KCNQ1* were highest in the fundic gland region. There were varying levels of expression of these genes in the three sampling sites of the cardiac gland region and little to no expression in the pyloric gland region. This pattern of expression for the acid-secreting genes is consistent with the spatial pattern of parietal cells in the stomach. Parietal cells are epithelial cells located predominantly in the fundic gland region of the stomach [15]. Their role is to release H^+^ and Cl^−^ ions into the stomach lumen, where the ions then associate to form HCl [24]. *ATP4A* encodes for the catalytic alpha subunit of the enzyme H^+^/K^+^-ATPase [25], which is unique to parietal cells and is involved in the active transport of H^+^ into the stomach in exchange for extracellular K^+^. *CLIC6* is the gene encoding a Cl^−^ apical channel protein. Ensuring that there is adequate luminal K^+^ concentration for this exchange to occur is a key part of the acid secretion process; in this sense, the recycling of K^+^ ions out of the parietal cell and back into the lumen is crucial. *KCNQ1* is a gene encoding for a potassium channel that is essential for acid secretion; it is suggested to secrete K^+^ into the lumen, where it can then be exchanged with H^+^ ions by H^+^/K^+^-ATPase [26,27]. The expression of these three genes was consistent across the three sampling sites of the fundic gland region sampled. In contrast, there was variation in expression in the cardiac gland region, where the expression of acid secretion genes increased from location 1 to location 3, with location 3 having the highest expression of *ATP4A* and *CLIC6*. This variation within the cardiac region is consistent with the “cardiac-to-oxyntic transition”. 

*AQP4* expression was highest in the fundic gland region, lower in the cardiac gland region and undetectable in the pyloric gland region. Aquaporin-4, encoded by the gene *AQP4*, is a water channel protein that is expressed in parietal cells, especially on the basolateral membrane [28]. It may play a role in the process of acid secretion, or it may simply act to add fluid to the base of the gland that aids in “washing out” the HCL into the lumen [29]. Arciszewski et al. 2015 reported that AQP4 was absent in all regions of the pig’s stomach by immunohistochemistry; however, similar to our observations, Colombo et al. reported expression of the gene *AQP4* in both the fundic and pyloric gland regions, with greater expression in the fundic gland region compared to the pyloric gland region [16,30]. Again, there was variation in the three sampling sites in the cardiac gland region with an increase in expression of *AQP4* from location 1 to location 3. 

Parietal cells are primarily stimulated by gastrin, histamine and acetylcholine. Gastrin, encoded by the gene *GAST*, is produced in G-cells and is the primary hormone that induces acid secretion from parietal cells and histamine secretion from ECL cells. In the current study, the pyloric gland region had the highest expression of *GAST*, while expression was undetectable in the cardiac gland region and undetectable in the majority of pigs in the fundic gland region. This aligns with observations from previous gene expression analysis studies [16] and spatial pattern analysis studies [15] in the stomach of the pig. Gastrin is secreted as the prohormone progastrin and undergoes post-translational processing to produce gastrin. The process is catalysed by prohormone convertase 1/3 (PC1/3), encoded by the gene *PCSK1* [31,32]. *PCSK1* expression was greater in the fundic and pyloric gland regions than in the cardiac gland region. The high expression in the fundic gland region is likely linked to the role of PC 1/3 in ghrelin processing [33], while the increased expression in the pyloric gland region is presumably due to its role in the processing of gastrin [32]. Once secreted, gastrin stimulates HCl release both directly, from parietal cells, and indirectly, via the stimulation of histamine release from the ECL cell. Gastrin stimulates acid secretion and histamine secretion by binding to the cholecystokinin B receptor (*CCKBR*) present on parietal and ECL cells, respectively [34,35,36]. Expression of *CCKBR* was high in the fundic gland region, low in the cardiac gland region and undetectable in the pyloric gland region. This agrees with the observed expression of genes associated with ECL and parietal cells, cells on which *CKKBR* is expressed [34,35,36]. There was no variation in the expression of *CCKBR* within the fundic or pyloric gland regions; however, similar to other genes associated with the fundic gland region, there was greater expression in location 3 compared to location 1 in the cardiac gland. 

Histamine stimulates parietal cells via the histamine receptor, encoded by the gene *HRH2* [37]. As expected, *HRH2* was similar in its pattern of expression to that of the other parietal-cell-related genes analysed in this study. *HRH2* expression was high in the fundic gland region, low in the cardiac gland region and extremely low in the pyloric gland region. There was no variation in the expression of *HRH2* within the fundic or pyloric gland regions; however, in the cardiac gland region, location 3 had greater expression relative to location 1. Histamine is predominantly synthesized in the stomach in enterochromaffin-like cells [38]. In these cells, histidine decarboxylase, encoded by the gene *HDC*, regulates the decarboxylation of histidine to produce histamine. Histamine acts in a paracrine fashion to stimulate acid secretion from the parietal cell via the *HRH2* receptor [39], suggesting that the ECL cells are located in close proximity to parietal cells, as is seen in other species such as guinea pigs [40], rats [41] and humans [42]. Surprisingly, Fothergill et al. reported that ECL cells were not located in proximity to parietal cells and were more abundant in the pyloric gland region than the fundic gland region [15]. In our current study, the *HDC* expression pattern is in opposition to that observed by Fothergill et al.; *HDC* expression was highest in the fundic gland region, with moderate to low expression in the cardiac gland region and minimal expression in the pyloric gland region [15]. This pattern of expression is similar to what was observed for genes related to acid secretion and suggests that the ECL cell, similar to what is seen in other species, is located close to the parietal cell, at least in terms of regional location in the stomach [40,41,42]. 

Somatostatin is a hormone secreted by D-cells that inhibits the secretion of acid by parietal cells [43], histamine from ECL cells [44], gastrin from G-cells and pepsin from chief cells [45]. In the current study, the expression of somatostatin, encoded by the gene *SST*, was highest in the pyloric gland region and remained reasonably high in both the cardiac and fundic gland regions, which is in agreement with what was observed previously in pigs [15,16]. This suggests that D-cells, although at their most abundant in the pyloric gland region, populate the entire stomach. This is in contrast to what is seen in humans, where the predominant location for D-cells is the fundic gland region rather than the pyloric gland region [46].

Ghrelin, encoded by the gene *GHRL*, is secreted by X/A-like cells and plays a role in many physiological functions involving hunger, feeding behaviour, motility of the GIT and energy balance [47,48,49,50,51]. In the present study, *GHRL* expression was highest in the fundic gland region, followed by the cardiac gland region and then the pyloric gland region. This agrees with what was observed in other studies in pigs [15,52,53]. Ghrelin is produced from the post-translational processing of preproghrelin. Preproghrelin is cleaved to produce proghrelin, which is then cleaved by ghrelin O-acetyltransferase (GOAT) and encoded by the gene *MBOAT4* to produce unacetylated ghrelin, its biologically inactive form, and obestatin [54]. Further processing of ghrelin is catalysed by the PC1/3 enzyme and encoded by the gene *PCSK1* [33,55]. The expression of *MBOAT4* was greater in the fundic gland region than the pyloric gland region, but it only tended to be greater in the fundic gland region compared to the cardiac gland region. Surprisingly, the cardiac gland region had a considerably high level of expression of *MBOAT4*, where there were low levels of *GHRL* expression. This is surprising, as in mice, the vast majority of GOAT-immunoreactive cells colabel with ghrelin [56,57]. *GHRL* and *MBOAT*4 had similar expression levels in the pyloric gland region. The significance of the high expression of *MBOAT4* in the cardiac gland region remains unclear, but it suggests that there may be an alternative exogenous substrate for this enzyme. 

Gastric chief cells are responsible for the secretion of gastric enzymes such as pepsinogen, chitinase and lipase [58,59,60,61]. Pepsinogen A, encoded by the gene *PGA5*, and acidic chitinase, encoded by the gene *CHIA*, are involved in the breakdown of protein and chitin, respectively. Chitin is one of the most abundant natural polysaccharides in nature and is a major biological component of fungi, crustaceans and insects [62]. Recently, the expression of the gene *CHIA* has been investigated in the stomach of the pig at different ages due to the growing interest in utilizing insects as an alternative protein source in pigs [63,64,65]. Gastric chief cells are highly expressed in the fundic gland region of the stomach [58,66]. In agreement with this, the expression of both *PGA5* and *CHIA* in the current study was highest in the fundic gland region with low expression in the cardiac gland region while expression was extremely low in the pyloric gland region. Cobalamin binding intrinsic factor, also known as gastric intrinsic factor, is an important digestive glycoprotein involved in vitamin B12 absorption. The expression of cobalamin binding intrinsic factor, encoded by the gene *CBLIF* (previously called *GIF*), was highest in the pyloric gland region, moderate in the fundic gland region and minimal in the cardiac gland region. This is in agreement with Colombo et al. and in contrast to that what is reported in humans and rodents [16]. In humans, cobalamin binding intrinsic factor is expressed in parietal cells, whereas in rodents it is expressed in chief cells deep in the fundic glands [67,68]. This suggests that there is an alternative cell in which cobalamin binding intrinsic factor is produced in pigs, as parietal and chief cells are extremely rare in the pyloric gland region [15,16]. The source of the high expression in the pyloric gland region remains unclear and requires further investigation. 

Mucins act as the structural component of the mucus that lines the stomach and protects the epithelial layer. Detailed research on the exact mucins present in the pig’s stomach is limited. The cardiac and pyloric glands are described as being dominated by mucus cells [15], although until now, it remained unclear whether these tissues had similar or unique mucin gene expression patterns. Padra et al. observed the presence of membrane associated *MUC1* and secreted mucins *MUC5AC*, *MUC6* and to some extent *MUC2* in the pyloric gland region [69]. Colombo et al. observed similar expression of the *MUC1* gene in the fundic and pyloric gland regions. In the current study, *MUC1* expression was high throughout the stomach. The fundic gland region had greater expression of *MUC1* than the cardiac gland region, and in agreement with Colombo et al., the fundic and pyloric gland regions had similar expression [16]. *MUC5AC* and *MUC6* expression was moderate in the cardiac gland region, with greater expression in the fundic and pyloric gland regions. The pattern of expression for *MUC2* was highly unique; it was highly expressed in the cardiac gland region and was undetectable in most pigs in the pyloric and fundic gland regions. *MUC2* expression tended to decrease from location 1 to location 3 in the cardiac gland region, another indication of the cardiac-to-oxyntic transition. To our knowledge, this is the first account of the variation in mucin gene expression patterns in the glandular regions of the pig’s stomach. 

Several genes involved in different components of the gastric mucosal defence system were analysed in this study: polymeric immunoglobulin receptor (pIgR), encoded by the gene *PIGR*; olfactomedin 4, encoded by the gene *OLFM4*; C-X-C motif chemokine ligand 8, encoded by the gene *CXCL8*; and toll-like receptors 2, 3, 4 and 5, encoded by the genes *TLR2*, *TLR3*, *TLR4* and *TLR5*, respectively. The pIgR mediates the transport of IgA from the basolateral surface of the epithelium to the apical side and releases it into the mucus layer. The releasing of the immunoglobulins into the mucus layer involves the cleavage of pIgR, releasing a component of its structure known as the secretory component (SC) along with the bound immunoglobulin, forming sIg. SC protects the immunoglobulin from degradation by enzymes [70]. Expression of *PIGR* has been observed to be greater in the pyloric gland region than in the fundic gland region [16,21]. The study conducted by Trevesi et al. also analysed the expression of *PIGR* in the cardiac-to-oxyntic transition, a region which is discussed further later in this paper, and observed similar expression levels of *PIGR* in this transition region as in the pyloric gland region [21]. In the current study, *PIGR* expression was highest in the cardiac gland region, where expression levels were very high. The pyloric gland region tended to have greater expression of *PIGR* compared to the fundic gland region. *OLFM4* expression was greater in the cardiac and pyloric compared to the fundic gland region. In the cardiac gland region, location 1 had greater expression of *PIGR* and *OLFM4* compared to location 3. C-X-C motif chemokine ligand 8 expression was greater in the cardiac gland region compared to the fundic or pyloric gland regions. The high expression of *OLFM4*, *PIGR* and *CXCL8* suggests that the cardiac gland region may play a role in immune functions in the stomach.

Some papers reference an area located between the cardiac and fundic gland regions, termed the cardiac-to-oxyntic transition, that contains a hybridlike tissue containing glands known as oxyntocardiac glands [21,71]. Although referenced in the literature, the precise location of this zone is not detailed. The cardiac gland region is described at a cellular level as being free from acid-producing parietal cells and enzyme-secreting chief cells. Therefore, the location at the cardiac gland region where the expression of acid secretion genes becomes upregulated should be considered the beginning of the cardiac-to-oxyntic transition. This transition zone is apparent in the differential expression evident between locations 1 and 3 in the cardiac gland region in the current study. Location 3 had greater expression of the acid secretion genes *ATP4A*, *CLIC6* and *HRH2*, parietal cell water transport gene *AQP4*, digestive enzyme gene *CHIA*, gastrin receptor gene *CCKBR* and cobalamin binding intrinsic factor *CBLIF*. In this study, the cardiac gland region is characterized by high expression of *PIGR* and *OLFM4*, which were downregulated in location 3 relative to location 1. From the gene expression data, the transition zone can be characterized by an increase in genes *ATP4A*, *CLIC6*, *HRH2*, *AQP4*, *CHIA*, *CCKBR* and *CBLIF* and a decrease in genes *OLFM4* and *PIGR* relative to the true cardiac gland region. There was also a tendency for a reduction in the *MUC*2 expression, a gene which is almost exclusively expressed in the true cardiac gland region, between location 1 and location 3. Interestingly, there was a negative correlation in the cardiac gland region between the acid secretion genes and the mucosal-barrier-related genes *PIGR*, *OLFM4* and *MUC2*. 

**Figure 5:**
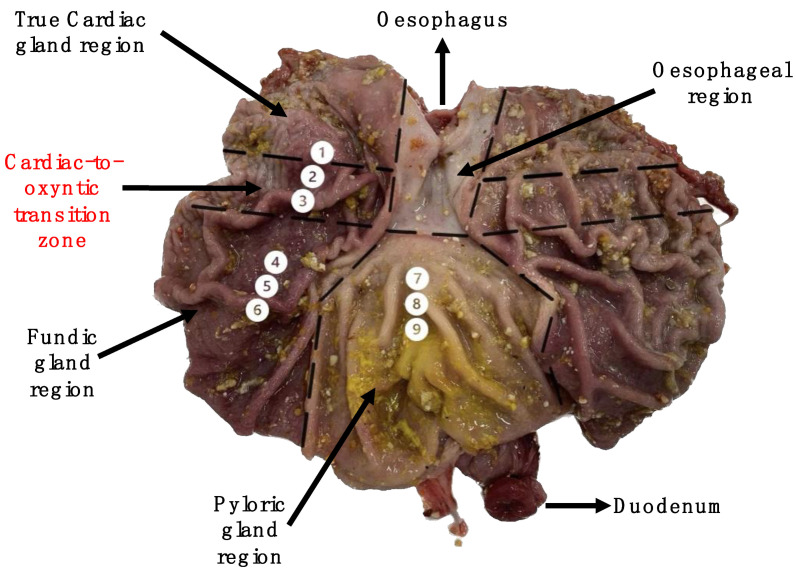
The approximate location of the cardiac-to-oxyntic transition. Mucosal sampling sites (1–9) are highlighted.

In the current study, in pigs where there was an expression of acid secretion genes in the cardiac gland region, expression in location 3 was always greater than in location 1. This indicates a transition gradient rather than discrete patches in the cardiac-to-oxyntic transition. There was also large variation observed at the location at which expression of acid secretion genes begins in the cardiac gland region. The reasons for this variation may reflect natural variation during embryonic development or environmental factors postnatally. While it could be argued that the precision of sampling technique used in the current study could contribute to some variation, particularly given the homogeneous nature of the region, the degree of variation observed suggests it cannot be the predominant cause. Based on the results of this study, the cardiac gland region can be subdivided into the “true cardiac gland region” and the “cardiac-to-oxyntic transition”. In the current study, location 1 is the most accurate representation of the true cardiac gland region, as the expression of acid-secretion-related genes is undetectable at this location in the majority pigs. To this extent, the diagram of the pig’s stomach has been revisited, and a guideline for the location of the true cardiac gland region and the cardiac-to-oxyntic transition has been added in Figure 5. 

Surprisingly, in a small number of fundic gland region samples, the expression of acid secretion genes was minimal or undetectable and there was a decrease in the expression of other fundic gland region associated genes such as ghrelin and digestive enzymes. In these samples, genes involved in mucosal defence, pro-inflammatory cytokine C-X-C motif ligand 8 and toll-like receptor 2 were increased. A similar observation was noted in the pyloric gland region, where expression of gastrin (*GAST)* was negatively correlated with C-X-C motif ligand 8 (*CXCL8*). In agreement with this, *CXCL8* expression was negatively correlated with the expression of *GAST*, *SST*, *CBLIF* and *PCSK1* in the pyloric gland region and *ATP4A*, *CHIA*, *AQP4*, *HRH2*, *KCNQ1*, *CCKBR*, *CLIC6*, *PCSK1*, *GHRL*, *HDC*, *PGA5* and *CBLIF* in the fundic gland region. *CXCL8* is an important pro-inflammatory cytokine. The fact that its expression negatively correlates with genes key to the functioning of the stomach highlights how inflammatory responses negatively influence a wide range of key cellular functions within the stomach. Strategies that help to modulate unregulated immune responses may also help to optimize the healthy function and promote the normal developmental trajectory of the stomach during the post-weaning period.

## 5. Conclusions

The present study characterized the variation in the expression of a panel of genes that play key roles in the functioning and regulation of the stomach. To our knowledge, this is the first study to characterize the expression pattern in the true cardiac gland region of the pig’s stomach while also revealing the potential size of the cardiac-to-oxyntic transition and the gene expression signature in this zone. The true cardiac gland region was characterized by a higher expression of *PIGR*, *OLFM4*, *CXCL8* and *MUC2* compared to the fundic and pyloric gland regions. This expression pattern suggests that the cardiac gland region may play a role in immune defence in the stomach. The cardiac-to-oxyntic transition was characterized by an increase in the expression of acid secretion (*ATP4A*, *CLIC6*, *HRH2*), *AQP4*, *CHIA*, *CCKBR* and *CBLIF*, with a decrease in expression of *PIGR* and *OLFM4* compared to the true cardiac gland region. The fundic gland region was characterized by high expression of acid secretion (*ATP4A*, *CLIC6*, *KCNQ1*, *HRH2*), *AQP4*, *HDC*, *CCKBR*, digestive enzyme (*CHIA*, *PGA5*) and ghrelin production (*GHRL*, *MBOAT4*) genes compared to the fundic and pyloric gland regions. The pyloric gland region was characterized by exclusive expression of *GAST* and a higher expression of *SST* and *CBLIF* compared to the cardiac and fundic gland regions. In conclusion, the glandular regions of the post-weaned pig’s stomach each have unique gene expression patterns, and the true cardiac gland region is located more proximally in the stomach than initially described in the literature.

## Figures and Tables

**Table 1:** Ingredients and chemical composition of the diet.

Ingredient	Quantity (g/kg)
Wheat	328.0
Barley	150.0
Full-fat soya bean	170.0
Soya bean meal	105.0
Whey powder (90%)	50.0
Soya oil	30.0
Soya concentrate	65.0
Maize	77.4
Lysine-HCl	4.0
Dl-methionine	2.0
L-threonine	1.8
Tryptophan	0.3
Sodium bicarbonate	2.0
Monocalcium phosphate	4.0
Mineral and vitamins	2.5
Calcium carbonate (limestone)	6.0
Salt	2.0
Composition	
Protein	207.0
Ether extract	83.0
Crude fibre	30.0
DE (MJ/kg)	15.0
NE (MJ/kg)	11.0
Lysine	14.7
SID lysine	13.3
Methionine + cysteine	9.0
Threonine	10.5
Tryptophan	3.0
Calcium	7.9
Total phosphorous	5.9

SID—standardised ileal digestible, DE—digestible energy, NE—net energy.

**Table 2:** Primer sequences for QPCR analysis.

Target Gene	Gene Name	Accession No.	Forward Primer (5‣-3‣) Reverse Primer (5‣-3‣)	Amplicon Length (bp)
Acid secretion				
*ATP4A*	ATPase H^+^/K^+^ Transporting Subunit Alpha	XM_021093570.1	F:GGACATGGCAGCCAAGATG R:TGTTCTCCAGCTTCTCCTTCCT	74
*CLIC6*	Chloride Intracellular Channel 6	XM_003358948.4	F:CGGAACCAGTCAGAAGAACGA R:TCCTACCGCCCAAGAAGCT	87
*HRH2*	Histamine Receptor 2	XM_003354192.4	F:CCAGCCTGGATGTCATGCCT R:CCGGTCGAGGCTGATCAT	65
*KCNQ1*	Potassium Voltage-Gated Channel Subfamily Q Member 1	XM_021082620.1	F:CGCGTCTACAACTTCCTCGAA R:CGATAAGGAAGACAGCAAAGTGGTA	73
Cobalamin binding intrinsic factor				
*CBLIF*	Cobalamin Binding Intrinsic Factor	XM_003122682.3	F:CGGAATCATTGGAAACATCTATAGC R:GGTCGCTCAGGTGTCACAGA	69
Digestive enzymes				
*CHIA*	Chitinase Acidic	NM_001258377.1	F:GCCTTTTGTACCCACCTGGTCTA R:TCAGTGGTGGTGATCTCGTTGT	65
*PGA5*	Pepsinogen A5	NM_213872.2	F:CGGCAGCGTGGTGGTGTTGT R:GGAAACAGGCACCCAGTTCA	73
Gastrin				
*GAST*	Gastrin	NM_001004036.2	F:TCCCAGCTCTGCAGTCAAGA R:CCAGAGCCAGCACATGGA	65
Gastrin receptor				
*CCKBR*	Cholecystokinin B Receptor	XM_021062350	F:CATGGGCACGTTTATCTTTGG R:TCACAGACACCCCCATGAAGT	68
Ghrelin production				
*GHRL*	Ghrelin	XM_013981924.2	F:AAGCTGGAAATCCGGTTCAA R:CGGACTGAGCCCCTGACA	64
*MBOAT4*	Membrane Bound O-Acyltransferase Domain Containing 4	NM_001190423.1	F:GCTCCCACCAAACCCAGA R:CCCACTGGATCCTGGATGAG	65
Histamine production				
*HDC*	Histidine Decarboxylase	XM_001925342.5	F:ATCTGCCAGTACCTGAGCACTGT R:GCAGGTAGCCAGGTCTCACATC	67
Inflammation				
CXCL8	C-X-C Motif Chemokine Ligand 8	NM_213867.1	F:TGCACTTACTCTTGCCAGAACTG R:CAAACTGGCTGTTGCCTTCTT	82
Mucins				
*MUC1*	Mucin 1	XM_001926883.1	F:ACACCCATGGGCGCTATGT R:GCCTGCAGAAACCTGCTCAT	68
*MUC2*	Mucin 2	AK231524	F:CAACGGCCTCTCCTTCTCTGT R:GCCACACTGGCCCTTTGT	70
*MUC5AC*	Mucin 5AC	XM_021092583.1	F:GGATGTCGCCAGAGACTGAGTA R:CCCCCTCGTCTCCTTTTACC	71
*MUC6*	Mucin 6	XM_021082474.1	F:AAAACGTGGGCAGGATGTGT R:GCCATCCTCGCTCAGAAACT	77
Mucosal defence				
*OLFM4*	Olfactomedin 4	XM_003482903.4	F:GGCGCCAGGGAGCTGTA R:TGAGTTGAACAATAGCCGGTTTG	65
*PIGR*	Polymeric Immunoglobulin Receptor	XM_021102216.1	F:GGGCTCGGTGACATTTGACT R:TTTAGCTGGCACAGAAATTTGG	72
Aquaporin water channel protein				
*AQP4*	Aquaporin 4	XM_021093195.1	F:GCAAAGCTAGCCAACAAACAAA R:CCTCGGTCTCAACCTGACTTCT	72
Prohormone processing				
*PCSK1*	Proprotein Convertase Subtilisin/Kexin Type 1	HE599222.1	F:GCAATTCTTTCTGGCTTTTCTACCT R:CACACTCGCCCGCATACA	72
Somatostatin				
*SST*	Somatostatin	NM_001009583.1	F:CCCTGGAGCCTGAAGATTTG R:GCCGGGTTTGAGTTAGCTGAT	85
Toll-like receptors				
*TLR2*	Toll-Like Receptor 2	NM_213761.1	F:CATCTTCGTGCTTTCCGAGAAC R:AAAGAGACGGAAGTGGGAGAAGT	79
*TLR3*	Toll-Like Receptor 3	NM_001097444.1	F:CATTGAGAATCTATCCCTGAGCAA R: TGCTGAGGTTTGTCTGCTTTAGTC	86
*TLR4*	Toll-Like Receptor 4	NM_001293317.1	F:TGCATGGAGCTGAATTTCTACAA R: GATAAATCCAGCACCTGCAGTTC	140
*TLR5*	Toll-Like Receptor 5	NM_001348771.1	F:CAGCCAGGCCGTCAATG R:AAGCCAAACCCAGAACCCATA	75
Reference genes				
*ACTB*	Beta Actin	XM_001927228.1	F:GGACATCGGATACCCAAGGA R:AAGTTGGAAGGCCGGTTAATTT	71
*B2M*	Beta-2-Microglobulin	NM_213978.1	F:CGGAAAGCCAAATTACCTGAAC R:TCTCCCCGTTTTTCAGCAAAT	83
*GAPDH*	Glyceraldehyde-3-Phosphate Dehydrogenase	AF017079.1	F:CAGCAATGCCTCCTGTACCA R:ACGATGCCGAAGTTGTCATG	72
*PPIA*	Peptidylprolyl Isomerase A	NM_214353.1	F:CGGGTCCTGGCATCTTGT R:TGGCAGTGCAAATGAAAAACT	75
*OAZ1*	Ornithine Decarboxylase Antizyme 1	NM_125342.1	F:CATCCCCTTGTCCCCAA R:ACCAGAGGACTCTCTCTCAAACGT	69
*RPS29*	Ribosomal Protein S29	NM_001001633.2	F:CGCATGCGTGCGCTAAG R:TGGTGACCCATCTTGCTCTCT	64
*RPL27*	Ribosomal Protein L27	NM_001097479.1	F:GTCCTGGCTGGTCGCTACTC R:GGTCTGAGGTGCCATCATCA	70
*RPL29*	Ribosomal Protein L29	XM_0023442034.1	F:GCCAATGTGAGGACAGAAGGA R:CAGGACACCAGCCCCGTATA	65

**Table 3:** Differential gene expression between the cardiac, fundic and pyloric regions (least squared means with their standard errors).

Function	Gene	Cardiac (C)	Fundic (F)	Pyloric (P)	SEM	*p*-Values		
						CF	CP	FP
Acid secretion	*ATP4A*	0.082	0.454	0.000	0.040	**<0.0001**	0.1540	**<0.0001**
	*CLIC6*	0.083	0.361	0.000	0.040	**<0.0001**	0.1428	**<0.0001**
	*HRH2*	0.096	0.402	0.033	0.037	**<0.0001**	0.2260	**<0.0001**
	*KCNQ1*	0.151	0.395	0.072	0.030	**<0.0001**	0.0689	**<0.0001**
Digestive enzyme production	*CHIA*	0.079	0.403	0.007	0.035	**<0.0001**	0.1552	**<0.0001**
	*PGA5*	0.008	0.152	0.013	0.031	**0.0018**	0.9106	**0.0026**
Gastrin production	*GAST*	0.000	0.002	0.212	0.020	0.9521	**<0.0001**	**<0.0001**
Gastrin receptor	*CCKBR*	0.053	0.317	0.000	0.034	**<0.0001**	0.2637	**<0.0001**
Ghrelin production	*GHRL*	0.254	0.442	0.131	0.036	**0.0004**	**0.0184**	**<0.0001**
	*MBOAT4*	0.286	0.363	0.146	0.029	0.0626	**0.0010**	**<0.0001**
Histamine production	*HDC*	0.120	0.338	0.040	0.029	**<0.0001**	0.0579	**<0.0001**
Inflammation	*CXCL8*	0.342	0.149	0.144	0.030	**<0.0001**	**<0.0001**	0.9065
Intrinsic factor production	*CBLIF*	0.059	0.227	0.475	0.037	**0.0020**	**<0.0001**	**<0.0001**
Mucosal defence	*PIGR*	0.523	0.165	0.263	0.040	**<0.0001**	**<0.0001**	0.0848
	*OLFM4*	0.368	0.139	0.276	0.043	**0.0004**	0.1364	**0.0286**
Mucus production	*MUC1*	0.298	0.388	0.343	0.030	**0.0341**	0.2771	0.2903
	*MUC2*	0.245	0.005	0.010	0.021	**<0.0001**	**<0.0001**	0.8760
	*MUC5AC*	0.145	0.360	0.435	0.034	**<0.0001**	**<0.0001**	0.1272
	*MUC6*	0.204	0.326	0.338	0.036	**0.0204**	**0.0113**	0.8206
Water channel protein	*AQP4*	0.076	0.427	0.000	0.042	**<0.0001**	0.2081	**<0.0001**
Prohormone processing	*PCSK1*	0.207	0.513	0.410	0.039	**<0.0001**	**0.0005**	0.0672
Somatostatin production	*SST*	0.166	0.240	0.562	0.034	0.1282	**<0.0001**	**<0.0001**
Toll-like receptor	*TLR2*	0.267	0.178	0.243	0.020	**0.0022**	0.3959	**0.0218**
	*TLR3*	0.323	0.550	0.621	0.028	**<0.0001**	**<0.0001**	0.0816
	*TLR4*	0.628	0.607	0.538	0.036	0.6854	0.0809	0.1722
	*TLR5*	0.107	0.481	0.442	0.024	**<0.0001**	**<0.0001**	0.2526

**Table 4:** Differentially enriched functions between the cardiac, fundic and pyloric regions (least squared means with their standard errors).

Function	Genes	Cardiac (C)	Fundic (F)	Pyloric (P)	SEM	*p*-Values		
						CF	CP	FP
Acid secretion	*ATP4A*, *KCNQ1*, *CLIC6*, *HRH2*	0.103	0.403	0.026	0.034	**<0.0001**	0.1159	**<0.0001**
Digestive enzymes	*CHIA*, *PGA5*	0.044	0.278	0.010	0.031	**<0.0001**	0.4531	**<0.0001**
Ghrelin production	*GHRL*, *MBOAT4*	0.270	0.403	0.139	0.028	**0.0015**	**0.0017**	**<0.0001**
Mucus production	*MUC1*, *MUC2*, *MUC5AC*, *MUC6*	0.223	0.270	0.281	0.018	0.0645	**0.0220**	0.6437
Toll-like receptors	*TLR2*, *TLR3*, *TLR4*, *TLR5*	0.331	0.454	0.461	0.0137	**<0.0001**	**<0.0001**	0.7216

**Table 5:** Differential expression between the three sampling locations within the cardiac region (least squared means with their standard errors).

Function	Gene	Location *			SEM	*p*-Values		
		1(a)	2(b)	3(c)		ab	ac	bc
Acid secretion	*ATP4A*	0.010	0.070	0.166	0.046	0.3693	**0.0263**	0.1559
	*CLIC6*	0.008	0.060	0.181	0.056	0.5178	**0.0410**	0.1436
	*HRH2*	0.030	0.095	0.164	0.043	0.2936	**0.0378**	0.2673
Digestive enzyme	*CHIA*	0.023	0.076	0.139	0.034	0.2756	**0.0246**	0.2071
	*PGA5*	0.004	0.008	0.014	0.004	0.5174	0.0937	0.2849
Gastrin receptor	*CCKBR*	0.008	0.040	0.110	0.033	0.4991	**0.0417**	0.1533
Histamine production	*HDC*	0.064	0.134	0.164	0.039	0.2199	0.0852	0.5935
Intrinsic factor	*CBLIF*	0.004	0.041	0.133	0.037	0.4800	**0.0222**	0.0947
Mucosal defence	*OLFM4*	0.485	0.406	0.213	0.086	0.5226	**0.0353**	0.1246
	*PIGR*	0.655	0.534	0.379	0.090	0.3492	**0.0407**	0.2345
Mucus production	*MUC2*	0.336	0.215	0.183	0.060	0.1657	0.0852	0.7150
Water channel protein	*AQP4*	0.001	0.069	0.154	0.045	0.3300	**0.0301**	0.1979
Prohormone processing	*PCSK1*	0.139	0.215	0.266	0.052	0.3103	0.0968	0.4924

* There was no differential expression within the fundic gland region (locations 4, 5 and 6) or within the pyloric gland region (locations 7, 8 and 9).

**Table 6:** Differentially enriched functions between the three sampling locations within the cardiac region (least squared means with their standard errors).

Role	Genes	Location *			SEM	*p*-Values		
		1(a)	2(b)	3(c)		ab	ac	bc
Acid secretion	*ATP4A*, *CLIC6*, *HRH2*, *KCNQ1*	0.045	0.091	0.173	0.038	0.4127	**0.0305**	0.1527
Digestive enzyme	*CHIA*, *PGA5*	0.013	0.042	0.076	0.019	0.2924	**0.0274**	0.2107

* There were no differentially enriched functions within the fundic gland region (locations 4, 5 and 6) or within the pyloric gland region (locations 7, 8 and 9).

## Data Availability

All data presented and/or analysed in this study are available on request from the corresponding author.

## Appendix A

**Table A1:** The least squared means of the expression of genes involved in acid secretion, digestive enzyme production, gastrin production, gastrin receptor, ghrelin production, inflammation, mucosal defence, mucus production, prohormone processing, somatostatin production and toll-like receptors per location in the stomach.

Function	Gene	Tissue									SEM		
		Cardiac (C)			Fundic (F)			Pyloric (P)			C	F	P
		1	2	3	4	5	6	7	8	9			
Acid secretion	*ATP4A*	0.0100	0.0700	0.1663	0.4763	0.4013	0.4838	0.0000	0.0000	0.0000	0.0462	0.1142	0.0000
	*HRH2*	0.0300	0.0950	0.1638	0.3850	0.3850	0.4363	0.0313	0.0288	0.0375	0.0426	0.1052	0.0036
	*CLIC6*	0.0075	0.0600	0.1813	0.4425	0.3125	0.3275	0.0000	0.0000	0.0000	0.0564	0.1049	0.0000
	*KCNQ1*	0.1338	0.1400	0.1800	0.4037	0.3650	0.4175	0.0588	0.0775	0.0788	0.0224	0.0912	0.0130
Digestive enzyme production	*CHIA*	0.0225	0.0763	0.1388	0.4188	0.3813	0.4087	0.0075	0.0063	0.0088	0.0339	0.1036	0.0025
	*PGA5*	0.0038	0.0075	0.0138	0.1688	0.1712	0.1162	0.0138	0.0125	0.0138	0.0040	0.0979	0.0021
Gastrin production	*GAST*	0.0000	0.0000	0.0000	0.0025	0.0000	0.0025	0.1713	0.2375	0.2263	0.0000	0.0002	0.0604
Gastrin receptor	*CCKBR*	0.0075	0.0400	0.1100	0.3912	0.2675	0.2913	0.0000	0.0000	0.0000	0.0334	0.0944	0.0000
Ghrelin production	*GHRL*	0.1875	0.2500	0.3238	0.4288	0.4100	0.4875	0.1263	0.1425	0.1250	0.0582	0.8652	0.0345
	*MBOAT4*	0.3163	0.2575	0.2838	0.3900	0.3625	0.3362	0.1363	0.1513	0.1500	0.0766	0.0417	0.0202
Intrinsic factor production	*CBLIF*	0.0038	0.0413	0.1325	0.2300	0.1913	0.2588	0.3563	0.5100	0.5575	0.0368	0.0635	0.0803
Histamine production	*HDC*	0.0637	0.1338	0.1638	0.4200	0.2950	0.2975	0.0350	0.0412	0.0437	0.0391	0.0788	0.0093
Inflammation	*CXCL8*	0.3600	0.3975	0.2687	0.1450	0.1725	0.1288	0.1588	0.1225	0.1600	0.0613	0.0554	0.0385
Mucosal defence	*PIGR*	0.6550	0.5338	0.3787	0.1750	0.1650	0.1538	0.2400	0.2550	0.2925	0.0895	0.0555	0.0494
	*OLFM4*	0.4850	0.4062	0.2125	0.1275	0.1638	0.1263	0.2338	0.2463	0.3475	0.0856	0.0728	0.0590
Mucus production	*MUC1*	0.3075	0.2875	0.2975	0.3513	0.3913	0.4213	0.3400	0.3462	0.3437	0.0477	0.0644	0.0455
	*MUC2*	0.3363	0.2150	0.1837	0.0100	0.0025	0.0025	0.0100	0.0125	0.0063	0.0597	0.0004	0.0004
	*MUC5AC*	0.1463	0.1388	0.1500	0.3500	0.3612	0.3687	0.4262	0.4350	0.4425	0.0112	0.0660	0.0837
	*MUC6*	0.2038	0.1937	0.2150	0.3425	0.2962	0.3388	0.3100	0.3225	0.3700	0.0422	0.0452	0.0946
Parietal cell water channel	*AQP4*	0.0050	0.0688	0.1538	0.4362	0.3888	0.4562	0.0000	0.0000	0.0000	0.0452	0.1219	0.0000
Prohormone processing	*PCSK1*	0.1388	0.2150	0.2663	0.5500	0.4750	0.5125	0.3325	0.4475	0.4487	0.0519	0.0679	0.0828
Somatostatin production	*SST*	0.1525	0.1675	0.1788	0.2725	0.2050	0.2425	0.4600	0.5762	0.6500	0.0259	0.0292	0.0937
Toll-like receptors	*TLR2*	0.2669	0.3214	0.2219	0.1890	0.2089	0.1757	0.2563	0.2276	0.2043	0.0521	0.0246	0.0172
	*TLR3*	0.2825	0.3037	0.3788	0.5640	0.5237	0.5592	0.6329	0.5789	0.6527	0.0531	0.0473	0.0472
	*TLR4*	0.6543	0.6567	0.5785	0.5805	0.5962	0.5644	0.6199	0.5314	0.5436	0.0668	0.0556	0.0685
	*TLR5*	0.0971	0.1024	0.1199	0.5138	0.4571	0.4678	0.3870	0.4447	0.5029	0.0149	0.0417	0.0550

## References

[1] Cranwell P.D. (1985). The development of acid and pepsin (EC 3. 4. 23. 1) secretory capacity in the pig; the effects of age and weaning: 1. Studies in anaesthetized pigs. Br. J. Nutr..

[2] Du G.M., Shi Z.M., Wei X.H., Liu M.J., Zhang L., Zhao R.Q. (2007). Expression of gastric ghrelin and H^+^–K^+^-ATPase MRNA in weanling piglets and effect of ghrelin on H^+^–K^+^-ATPase expression and activity in gastric mucosal cells in vitro. Res. Vet. Sci..

[3] Trevisi P., Luise D., Correa F., Messori S., Mazzoni M., Lallès J.P., Bosi P. (2021). Maternal antibiotic treatment affects offspring gastric sensing for umami taste and ghrelin regulation in the pig. J. Anim. Sci. Biotechnol..

[4] Barrow P., Fuller R., Newport M. (1977). Changes in the microflora and physiology of the anterior intestinal tract of pigs weaned at 2 days, with special reference to the pathogenesis of diarrhea. Infect. Immun..

[5] Cranwell P., Noakes D., Hill K. (1976). Gastric secretion and fermentation in the suckling pig. Br. J. Nutr..

[6] Suiryanrayna M.V.A.N., Ramana J.V. (2015). A review of the effects of dietary organic acids fed to swine. J. Anim. Sci. Biotechnol..

[7] Lawlor P.G., Lynch P.B., Caffrey P.J., O’Reilly J.J., O’Connell M.K. (2005). Measurements of the acid-binding capacity of ingredients used in pig diets. Ir. Vet. J..

[8] Lawlor P.G., Lynch P.B., Caffrey P.J. (2006). Effect of Fumaric Acid, Calcium Formate and Mineral Levels in Diets on the Intake and Growth Performance of Newly Weaned Pigs. Ir. J. Agric. Food Res..

[9] Hedemann M.S., Jensen B.B. (2004). Variations in enzyme activity in stomach and pancreatic tissue and digesta in piglets around weaning. Arch. Anim. Nutr..

[10] Luise D., Motta V., Salvarani C., Chiappelli M., Fusco L., Bertocchi M., Mazzoni M., Maiorano G., Costa L.N., Van Milgen J. (2017). Long-term administration of formic acid to weaners: Influence on intestinal microbiota, immunity parameters and growth performance. Anim. Feed. Sci. Technol..

[11] Colombo M., Priori D., Gandolfi G., Boatto G., Nieddu M., Bosi P., Trevisi P. (2014). Effect of free thymol on differential gene expression in gastric mucosa of the young pig. Animal.

[12] Mazzoni M., Le Gall M., De Filippi S., Minieri L., Trevisi P., Wolinski J., Lalatta-Costerbosa G., Lallès J.-P., Guilloteau P., Bosi P. (2008). Supplemental Sodium Butyrate Stimulates Different Gastric Cells in Weaned Pigs. J. Nutr..

[13] Bosi P., Mazzoni M., De Filippi S., Trevisi P., Casini L., Petrosino G., Lalatta-Costerbosa G. (2006). A continuous dietary supply of free calcium formate negatively affects the parietal cell population and gastric RNA expression for H^+^/K^+^-ATPase in weaning pigs. J. Nutr..

[14] Yin J., Li X., Li D., Yue T., Fang Q., Ni J., Zhou X., Wu G. (2009). Dietary supplementation with zinc oxide stimulates ghrelin secretion from the stomach of young pigs. J. Nutr. Biochem..

[15] Fothergill L.J., Galiazzo G., Hunne B., Stebbing M.J., Fakhry J., Weissenborn F., Fazio Coles T.E., Furness J.B. (2019). Distribution and co-expression patterns of specific cell markers of enteroendocrine cells in pig gastric epithelium. Cell Tissue Res..

[16] Colombo M., Priori D., Trevisi P., Bosi P. (2014). Differential Gene Expression in the Oxyntic and Pyloric Mucosa of the Young Pig. PLoS ONE.

[17] Mazzoni M., Bosi P., De Sordi N., Lalatta-Costerbosa G. (2011). Distribution, organization and innervation of gastric MALT in conventional piglet. J. Anat..

[18] Gabriel G.C., Devine W.A., Redel B.K., Whitworth K.M., Samuel M., Spate L.D., Cecil R.F., Prather R.S., Wu Y.L., Wells K.D. (2022). Profiling development of abdominal organs in the pig. Sci. Rep..

[19] McCracken K.W., Wells J.M. (2017). Mechanisms of embryonic stomach development. Semin. Cell Dev. Biol..

[20] Xian Y., Zhao X., Wang C., Kang C., Ding L., Zhu W., Hang S. (2018). Phenylalanine and tryptophan stimulate gastrin and somatostatin secretion and H^+^-K^+^-ATPase activity in pigs through calcium-sensing receptor. Gen. Comp. Endocrinol..

[21] Trevisi P., Gandolfi G., Priori D., Messori S., Colombo M., Mazzoni M., Lallès J.-P., Bosi P. (2013). Age-Related Expression of the Polymeric Immunoglobulin Receptor (pIgR) in the Gastric Mucosa of Young Pigs. PLoS ONE.

[22] Wei T., Simko V., Levy M., Xie Y., Jin Y., Zemla J. (2017). Package ‘corrplot’. Statistician.

[23] R Core Team (2013). R: A Language and Environment for Statistical Computing.

[24] Yao X., Forte J.G. (2003). Cell biology of acid secretion by the parietal cell. Annu. Rev. Physiol..

[25] Shin J.M., Munson K., Vagin O., Sachs G. (2009). The gastric HK-ATPase: Structure, function, and inhibition. Pflügers Arch. -Eur. J. Physiol..

[26] Lambrecht N.W.G., Yakubov I., Scott D., Sachs G. (2005). Identification of the K efflux channel coupled to the gastric H-K-ATPase during acid secretion. Physiol. Genom..

[27] Grahammer F., Wittekindt O.H., Nitschke R., Herling A.W., Lang H.J., Bleich M., Schmitt-Gräff A., Barhanin J., Warth R. (2001). The cardiac K^+^ channel KCNQ1 is essential for gastric acid secretion. Gastroenterology.

[28] Fujita A., Horio Y., Nielsen S., Nagelhus E.A., Hata F., Ottersen O.P., Kurachi Y. (1999). High-resolution immunogold cytochemistry indicates that AQP4 is concentrated along the basal membrane of parietal cell in rat stomach. FEBS Lett..

[29] Wang K.S., Komar A.R., Ma T., Filiz F., McLeroy J., Hoda K., Verkman A.S., Bastidas J.A. (2000). Gastric acid secretion in aquaporin-4 knockout mice. Am. J. Physiol. -Gastrointest. Liver Physiol..

[30] Arciszewski M.B., Matysek M., Sienkiewicz W. (2015). Immunohistochemical localization of aquaporin 4 (AQP4) in the porcine gastrointestinal tract. Acta Vet. Brno.

[31] Sawada M., Finniss S., Dickinson C.J. (2000). Diminished prohormone convertase 3 expression (PC1/PC3) inhibits progastrin post-translational processing. Regul. Pept..

[32] Rehfeld J.F., Zhu X., Norrbom C., Bundgaard J.R., Johnsen A.H., Nielsen J.E., Vikesaa J., Stein J., Dey A., Steiner D.F. (2008). Prohormone convertases 1/3 and 2 together orchestrate the site-specific cleavages of progastrin to release gastrin-34 and gastrin-17. Biochem. J..

[33] Cleverdon E.R., McGovern-Gooch K.R., Hougland J.L. (2017). The octanoylated energy regulating hormone ghrelin: An expanded view of ghrelin’s biological interactions and avenues for controlling ghrelin signaling. Mol. Membr. Biol..

[34] Kopin A.S., Lee Y.-M., McBride E.W., Miller L.J., Lu M., Lin H.Y., Kolakowski L.F., Beinborn M. (1992). Expression cloning and characterization of the canine parietal cell gastrin receptor. Proc. Natl. Acad. Sci. USA.

[35] Nakata H., Matsui T., Ito M., Taniguchi T., Naribayashi Y., Arima N., Nakamura A., Kinoshita Y., Chihara K., Hosoda S. (1992). Cloning and characterization of gastrin receptor from ECL carcinoid tumor of Mastomys natalensis. Biochem. Biophys. Res. Commun..

[36] Kulaksiz H., Arnold R., Göke B., Maronde E., Meyer M., Fahrenholz F., Forssmann W.-G., Eissele R. (2000). Expression and cell-specific localization of the cholecystokinin B/gastrin receptor in the human stomach. Cell Tissue Res..

[37] Samuelson L.C., Hinkle K.L. (2003). Insights into the regulation of gastric acid secretion through analysis of genetically engineered mice. Annu. Rev. Physiol..

[38] Contreras-Aguilar M.D., López-Arjona M., Martínez-Miró S., Escribano D., Hernández-Ruipérez F., Cerón J.J., Tecles F. (2021). Changes in saliva analytes during pregnancy, farrowing and lactation in sows: A sialochemistry approach. Vet. J..

[39] Soll A., Walsh J.H. (1979). Regulation of gastric acid secretion. Annu. Rev. Physiol..

[40] Kamoshida S., Saito E., Fukuda S., Kato K., Iwasaki A., Arakawa Y. (1999). Anatomical location of enterochromaffin-like (ECL) cells, parietal cells, and chief cells in the stomach demonstrated by immunocytochemistry and electron microscopy. J. Gastroenterol..

[41] Hunne B., Stebbing M.J., McQuade R.M., Furness J.B. (2019). Distributions and relationships of chemically defined enteroendocrine cells in the rat gastric mucosa. Cell Tissue Res..

[42] Fakhry J., Stebbing M.J., Hunne B., Bayguinov Y., Ward S.M., Sasse K.C., Callaghan B., McQuade R.M., Furness J.B. (2019). Relationships of endocrine cells to each other and to other cell types in the human gastric fundus and corpus. Cell Tissue Res..

[43] Raptis S., Dollinger H., Von Berger L., Schlegel W., Schröder K., Pfeiffer E. (1975). Effects of somatostatin on gastric secretion and gastrin release in man. Digestion.

[44] Prinz C., Kajimura M., Scott D.R., Mercier F., Helander H.F., Sachs G. (1993). Histamine secretion from rat enterochromaffinlike cells. Gastroenterology.

[45] D’sa A.B., Bloom S., Baron J. (1978). Inhibition by somatostatin (growth-hormone release-inhibiting hormone, GH-RIH) of gastric acid and pepsin and G-cell release of gastrin. Gut.

[46] Choi E., Roland J.T., Barlow B.J., O’Neal R., Rich A.E., Nam K.T., Shi C., Goldenring J.R. (2014). Cell lineage distribution atlas of the human stomach reveals heterogeneous gland populations in the gastric antrum. Gut.

[47] Vizcarra J.A., Kirby J.D., Kim S.K., Galyean M.L. (2007). Active immunization against ghrelin decreases weight gain and alters plasma concentrations of growth hormone in growing pigs. Domest. Anim. Endocrinol..

[48] Barretero-Hernandez R., Galyean M.L., Vizcarra J.A. (2010). The Effect of Feed Restriction on Plasma Ghrelin, Growth Hormone, Insulin, and Glucose Tolerance in Pigs. Prof. Anim. Sci..

[49] Zhang H., Yin J., Li D., Zhou X., Li X. (2007). Tryptophan enhances ghrelin expression and secretion associated with increased food intake and weight gain in weanling pigs. Domest. Anim. Endocrinol..

[50] Dong X.-Y., Xu J., Tang S.-Q., Li H.-Y., Jiang Q.-Y., Zou X.-T. (2009). Ghrelin and its biological effects on pigs. Peptides.

[51] Scrimgeour K., Gresham M.J., Giles L.R., Thomson P.C., Wynn P.C., Newman R.E. (2008). Ghrelin secretion is more closely aligned to energy balance than with feeding behaviour in the grower pig. J. Endocrinol..

[52] Govoni N., De Iasio R., Cocco C., Parmeggiani A., Galeati G., Pagotto U., Brancia C., Spinaci M., Tamanini C., Pasquali R. (2005). Gastric immunolocalization and plasma profiles of acyl-ghrelin in fasted and fasted-refed prepuberal gilts. J. Endocrinol..

[53] Priori D., Trevisi P., Mazzoni M., Messori S., Bosi P. (2015). Effect of fasting and refeeding on expression of genes involved in the gastric nutrient sensing and orexigenic control of pigs. J. Anim. Physiol. Anim. Nutr..

[54] Zhang J.V., Ren P.G., Avsian-Kretchmer O., Luo C.W., Rauch R., Klein C., Hsueh A.J. (2005). Obestatin, a peptide encoded by the ghrelin gene, opposes ghrelin’s effects on food intake. Science.

[55] Steinert R.E., Feinle-Bisset C., Geary N., Beglinger C. (2013). Digestive physiology of the pig symposium: Secretion of gastrointestinal hormones and eating control. J. Anim. Sci..

[56] Stengel A., Goebel M., Wang L., Taché Y., Sachs G., Lambrecht N.W.G. (2010). Differential distribution of ghrelin-O-acyltransferase (GOAT) immunoreactive cells in the mouse and rat gastric oxyntic mucosa. Biochem. Biophys. Res. Commun..

[57] Sakata I., Yang J., Lee C.E., Osborne-Lawrence S., Rovinsky S.A., Elmquist J.K., Zigman J.M. (2009). Colocalization of ghrelin O-acyltransferase and ghrelin in gastric mucosal cells. Am. J. Physiol. Endocrinol. Metab..

[58] Moreau H., Bernadac A., Gargouri Y., Benkouka F., Laugier R., Verger R. (1989). Immunocytolocalization of human gastric lipase in chief cells of the fundic mucosa. Histochemistry.

[59] Ohno M., Kimura M., Miyazaki H., Okawa K., Onuki R., Nemoto C., Tabata E., Wakita S., Kashimura A., Sakaguchi M. (2016). Acidic mammalian chitinase is a proteases-resistant glycosidase in mouse digestive system. Sci. Rep..

[60] Goto M., Fujimoto W., Nio J., Iwanaga T., Kawasaki T. (2003). Immunohistochemical demonstration of acidic mammalian chitinase in the mouse salivary gland and gastric mucosa. Arch. Oral Biol..

[61] Suzuki M., Fujimoto W., Goto M., Morimatsu M., Syuto B., Iwanaga T. (2002). Cellular expression of gut chitinase mRNA in the gastrointestinal tract of mice and chickens. J. Histochem. Cytochem..

[62] Peniche C., Argüelles-Monal W., Goycoolea F.M., Belgacem M.N., Gandini A. (2008). Chapter 25-Chitin and Chitosan: Major Sources, Properties and Applications. Monomers, Polymers and Composites from Renewable Resources.

[63] Hong J., Kim Y.Y. (2022). Insect as feed ingredients for pigs. Anim. Biosci..

[64] Kawasaki K., Osafune T., Tamehira S., Yano K. (2021). Piglets can secrete acidic mammalian chitinase from the pre weaning stage. Sci. Rep..

[65] Tabata E., Kashimura A., Wakita S., Ohno M., Sakaguchi M., Sugahara Y., Imamura Y., Seki S., Ueda H., Matoska V. (2017). Protease resistance of porcine acidic mammalian chitinase under gastrointestinal conditions implies that chitin-containing organisms can be sustainable dietary resources. Sci. Rep..

[66] Ghoshal N.G., Bal H.S. (1989). Comparative morphology of the stomach of some laboratory mammals. Lab. Anim..

[67] Dieckgraefe B.K., Seetharam B., Alpers D.H. (1988). Developmental regulation of rat intrinsic factor mRNA. Am. J. Physiol. Gastrointest. Liver Physiol..

[68] O’Neil A., Petersen C.P., Choi E., Engevik A.C., Goldenring J.R. (2017). Unique Cellular Lineage Composition of the First Gland of the Mouse Gastric Corpus. J. Histochem. Cytochem..

[69] Padra M., Adamczyk B., Benktander J., Flahou B., Skoog E.C., Padra J.T., Smet A., Jin C., Ducatelle R., Samuelsson T. (2018). Helicobacter suis binding to carbohydrates on human and porcine gastric mucins and glycolipids occurs via two modes. Virulence.

[70] Duc M., Johansen F.-E., Corthésy B. (2010). Antigen binding to secretory immunoglobulin A results in decreased sensitivity to intestinal proteases and increased binding to cellular Fc receptors. J. Biol. Chem..

[71] Kim A., Park W.Y., Shin N., Lee H.J., Kim Y.K., Lee S.J., Hwang C.S., Park D.Y., Kim G.H., Lee B.E. (2015). Cardiac mucosa at the gastroesophageal junction: An Eastern perspective. World J. Gastroenterol..

